# Synthesis and antibacterial activity of novel levofloxacin derivatives containing a substituted thienylethyl moiety

**DOI:** 10.1186/2008-2231-20-16

**Published:** 2012-08-30

**Authors:** Negar Mohammadhosseini, Zahra Alipanahi, Eskandar Alipour, Saeed Emami, Mohammad Ali Faramarzi, Nasrin Samadi, Nika Khoshnevis, Abbass Shafiee, Alireza Foroumadi

**Affiliations:** 1 Department of Chemistry, North Tehran Branch, Islamic Azad University, Tehran, Iran; 2 Department of Medicinal Chemistry, Faculty of Pharmacy and Pharmaceutical Sciences Research Center, Tehran University of Medical Sciences, Tehran, Iran; 3 Department of Medicinal Chemistry and Pharmaceutical Sciences Research Center, Faculty of Pharmacy, Mazandaran University of Medical Sciences, Sari, Iran; 4 Department of Pharmaceutical Biotechnology, Faculty of Pharmacy, Tehran University of Medical Sciences, Tehran, Iran

**Keywords:** Antibacterial activity, Quinolones, Levofloxacin, Thiophene derivatives, Oximes

## Abstract

**Background and the purpose of the study:**

Piperazinyl quinolones such as ciprofloxacin, ofloxacin and levofloxacin are an important group of quinolone antimicrobials which are widely used in the treatment of various infectious diseases. In the present study, we synthesized a new series of levofloxacin derivatives and evaluated their antibacterial activities.

**Methods:**

The *N*-substituted analogs of levofloxacin **6a–j** were prepared by nucleophilic reaction of *N*-desmethyl levofloxacin **11** with thienylethyl bromide derivatives **8** or **9**. All target compounds were tested using conventional agar dilution method in comparison to levofloxacin and *N*-desmethyl levofloxacin and their MIC values were determined against a panel of Gram-positive and Gram-negative bacteria.

**Results:**

All compounds showed significant antibacterial activities against Gram-positive bacteria (MIC = 0.04-6.25 μg/mL); however, the activity against Gram-negative bacteria was lower (MIC = 1.56–100 μg/mL). As is evident from the data, oxime derivatives **6e**, **6h** and **6i** are superior in inhibiting the growth of Gram-positive bacteria (MIC = 0.04–0.19 μg/mL), and their activities were found to be 5–25 times better than *N*-desmethyl levofloxacin **11** and equal or better than levofloxacin **4**.

**Conclusion:**

We have designed and synthesized novel quinolone derivatives bearing functionalized thienylethyl moiety on the piperazine ring of levofloxacin. The results of antibacterial screening against Gram-positive and Gram-negative bacteria revealed that the introduction of functionalized thienylethyl moiety on the piperazine ring of levofloxacin can improve the activity against Gram-positive bacteria. Gram-positive bacteria are responsible for a wide range of infectious diseases, and rising resistance in this group is causing increasing concern. Thus, this study introduces structural features of levofloxacin scaffold for development of new candidates in the field of anti-Gram positive chemotherapy

## Introduction

**Figure 1:**
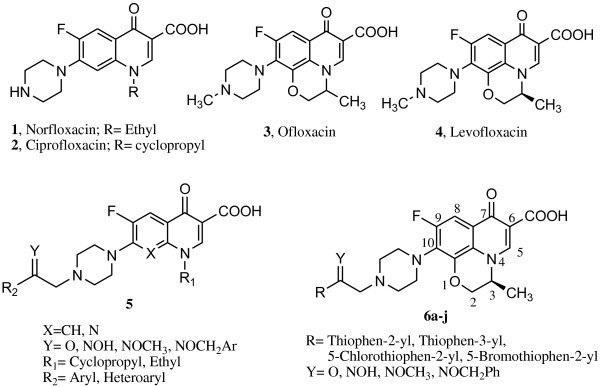
The structures of some known fluoroquinolones (1-5) and the synthesized compounds 6a-j.

Fluoroquinolones which are synthetic antibacterial agents are useful for the treatment of urinary tract infections, soft tissue infections, respiratory infections, typhoid fever, sexually transmitted diseases, bone-joint infections, community-acquired pneumonia, acute bronchitis, and sinusitis [1]. Fluoroquinolones consist of a bicyclic ring structure in which there is a substitution at position *N*-1; most of the current agents have a carboxyl group at position 3, a keto group at position 4, a fluorine atom at position 6 and a nitrogen heterocyclic moiety at the C-7 position [2]. Piperazine substitution at C-7 position has resulted in a wide range of clinically useful fluoroquinolone antibacterial agents namely norfloxacin (**1**), ciprofloxacin (**2**), ofloxacin (**3**) and levofloxacin (**4**) [3,4]. Fluoroquinolones with 7-piperazinyl moiety have been reported to possess potent antibacterial activity [5,6]. Levofloxacin, a chiral version of the earlier drug ofloxacin, is a successful fluoroquinolone antibacterial that departs from the typical quinolones substructure by having a fused ring connecting the N_1_ to C_8_ position [7]. This ring is connected with an ether moiety at C_8_[8]. Levofloxacin has been reported to be active in vitro against both Gram-positive and Gram-negative bacteria. Two major targets have been identified for levofloxacin activity, topoisomerase II (DNA-gyrase) in Gram-negative bacteria and topoisomerase IV in Gram-positive bacteria [9]. According to the inhibition mechanisms of the quinolones, the site near the C-7 substituent is regarded as drug-enzyme interaction domain and controls the spectrum and selectivity of quinolone molecule [10]. Over the past few years, we were particularly interested in the synthesis and evaluation of *N*-substituted 7-piperazinyl quinolones [5] for their antibacterial activities [11-16]. Although the nature of the C-7 substituent was known to influence quinolone activity in bacteria, we identified that addition of a certain bulky group as a particular chemical modification is permitted at the C-7 position of the piperazine ring and allows manipulation of selectivity and potency. Previously, we described the synthesis and antibacterial activity of certain piperazinyl quinolones (Figure 1), with 2-(thiophen-3-yl)-2-oxoethyl or 2-(thiophen-3-yl)-2-oxyiminoethyl moieties attached to the piperazine ring at the C-7 position [12]. In the present study, we have aimed to achieve new quinolone antibacterial, by preparing the levofloxacin derivatives **6a–j** carrying a 2-thienyl-2-oxoethyl or a 2-thienyl-2-oxyiminoethyl moiety attached to the piperazine ring at C-10 position (Figure 1).

## Material and methods

### Chemistry

**Figure 2:**
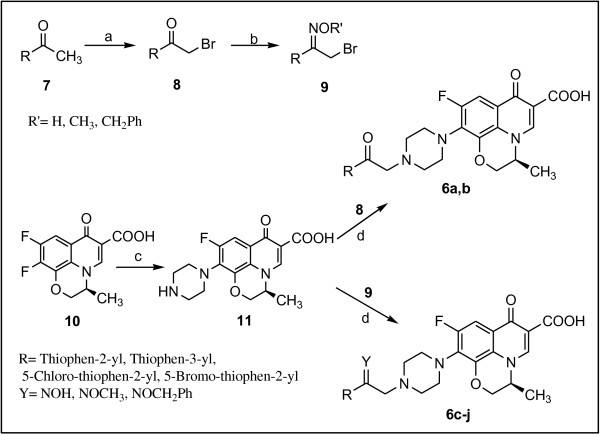
**Synthesis of levofloxacin derivatives 6a–j. ***Reagents and conditions* :(**a**) CuBr_2_, CHCl_3_–EtOAc, reflux;(**b**) HONH_2_·HCl orMeONH_2_·HCl or BnONH_2_·HCl, MeOH, r.t.; (**c**) piperazine; (**d**) DMF, NaHCO_3_, r.t.

Chemicals and all solvents used in this study were purchased from Merck AG and Aldrich Chemical. Melting points were determined on a Kofler hot stage apparatus and are uncorrected. The IR spectra were obtained on a Shimadzu 470 spectrophotometer (potassium bromide disks). ^1^H-NMR spectra were measured using a Bruker 400 spectrometer, and chemical shifts are expressed as δ (ppm) with tetramethylsilane as internal standard. Merck silica gel 60 F254 plates were used for analytical TLC. Yields are of purified product and were not optimized. The intermediate compounds **9** were prepared according to the literature [12,14]. *N*-Desmethyl levofloxacin was prepared as previously described method [17]. The synthetic route for achieving the target compounds **6a–j** was depicted in Figure 2.

### General procedure for the synthesis of compounds **6a–j**

A mixture of thienylethyl bromide derivatives **8** or **9** (0.55 mmol), *N*-desmethyl levofloxacin **11** (174 mg, 0.5 mmol) and NaHCO_3_ (42 mg, 0.5 mmol) in DMF (5 mL) was stirred at room temperature for 6–10 h. After consumption of *N*-desmethyl levofloxacin **11** (monitored by TLC), water (20 mL) was added and the precipitate was filtered, washed with water and crystallized from methanol-chloroform (9:1) to give compound **6a–j**.

#### *10-(4-(2-(5-bromothiophen-2-yl)-2-oxoethyl)piperazin-1-yl)-9-fluoro-3-methyl-7-oxo-3,7-dihydro-2H-[1,4]oxazino[2,3,4-Ij]quinoline-6-Carboxylic acid (**6a**)*

Yield: 31%; mp 165–166°C; IR (KBr) cm^−1^: 1622, 1721 (C = O). ^1^H-NMR (DMSO-*d*_6_): 1.45 (d, 3H, CH_3_, *J* = 6.0 Hz), 3.26–3.33 (m, 4H, piperazine), 3.38–3.42 (m, 4H, piperazine), 3.49 (s, 2H, CH_2_), 3.75 (s, 1H, CH_2_), 4.39 (d, 1H, CH_2_, *J* = 10.9 Hz), 4.60 (d, 1H, CH_2_, *J* = 10.9 Hz), 4.87 (m, 1H, CH-Me), 7.53 (d, 1H, thiophene, *J* = 5.0 Hz), 7.58–7.65 (m, 1H, thiophene), 7.81 (d, 1H, H_8_, *J* = 11.9 Hz), 8.99 (s, 1H, H_5_), 15.17 (s, 1H, COOH).

#### *10-(4-(2-(5-chlorothiophen-2-yl)-2-oxoethyl)piperazin-1-yl)-9-fluoro-3-methyl-7-oxo-3,7-dihydro-2H-[1,4]oxazino[2,3,4-Ij]quinoline-6-Carboxylic acid (**6b**)*

Yield: 26%; mp 156–157°C; IR (KBr) cm^−1^: 1620, 1719 (C = O). ^1^H-NMR (DMSO-*d*_6_): 1.43 (d, 3H, CH_3_, *J* = 6.2 Hz), 3.27–3.34 (m, 4H, piperazine), 3.36–3.41 (m, 4H, piperazine), 3.47 (s, 2H, CH_2_), 3.77 (s, 1H, CH_2_), 4.40 (d, 1H, CH_2_, *J* = 11.0 Hz), 4.59 (d, 1H, CH_2_, *J* = 11.0 Hz), 4.85 (m, 1H, CH-Me), 7.52 (d, 1H, thiophene, *J* = 5.0 Hz), 7.56–7.64 (m, 1H, thiophene), 7.79 (d, 1H, H_8_, *J* = 12.1 Hz), 8.98 (s, 1H, H_5_), 15.21 (s, 1H, COOH).

#### *10-(4-(2-(5-chlorothiophen-2-yl)-2-(methoxyimino)ethyl)piperazin-1-yl)-9-fluoro-3-methyl-7-oxo-3,7-dihydro-2H-[1,4]oxazino[2,3,4-Ij]quinoline-6-Carboxylic acid (**6c**)*

Yield: 24%; mp 180–181°C; IR (KBr) cm^−1^: 1626, 1717 (C = O). ^1^H-NMR (DMSO-*d*_6_): 1.62 (d, 3H, CH_3_, *J* = 5.3 Hz), 2.47–2.70 (m, 4H, piperazine), 3.34–3.42 (m, 4H, piperazine), 3.51 and 3.96 (two s, 2H, CH_2_, *E*- and *Z*-isomers), 4.04 and 4.07 (two s, 3H, OMe, *E*- and *Z*-isomers), 4.31–4.37 (m, 1H, CH_2_), 4.42–4.52 (m, 1H, CH_2_), 4.47–4.51 (m, 1H, CH-Me), 7.04 and 7.08 (two d, 1H, thiophene, *E*- and *Z*-isomers, *J* = 4.0 Hz), 7.63 and 7.69 (two d, 1H, thiophene, *E*- and *Z*-isomers, *J* = 4.0 Hz), 7.73 (d, 1H, H_8_, *J* = 12.3 Hz), 8.61 (s, 1H, H_5_), 15.01 (s, 1H, COOH).

#### *10-(4-(2-(benzyloxyimino)-2-(5-chlorothiophen-2-yl) ethyl) piperazin-1-yl)-9-fluoro-3-methyl-7-oxo-3, 7-dihydro-2H-[1, 4]oxazino [2, 3, 4-ij]quinoline-6-carboxylic acid (**6d**)*

Yield: 15%; mp 143–144°C; IR (KBr) cm^−1^: ^1^H-NMR (DMSO-*d*_6_): 1.62 (d, 3H, CH_3_, *J* = 6.6 Hz), 2.47–2.72 (m, 4H, piperazine), 3.28–3.43 (m, 4H, piperazine), 3.50 and 3.65 (two s, 2H, CH_2_, *E*- and *Z*-isomers), 4.34 (d, 1H, CH_2_, *J* = 6.6 Hz), 4.47 (d, 1H, CH_2_, *J* = 6.6 Hz), 5.19 and 5.26 (two s, 2H, OCH_2_, *E*- and *Z*-isomers), 5.32 (s, 1H, CH-Me), 6.82 and 6.84 (two d, 1H, thiophene, *E*- and *Z*-isomers, *J* = 4.0 Hz), 6.91 and 6.94 (two d, 1H, thiophene, *E*- and *Z*-isomers, *J* = 4.0 Hz), 7.36–7.40 (m, 5H, phenyl), 7.74 (d, 1H, H_8_, *J* = 10.0 Hz), 8.61(s, 1H, H_5_), 15.00 (s, 1H, COOH).

#### *9-fluoro-10-(4-(2-(hydroxyimino)-2-(thiophen-2-yl)ethyl)piperazin-1-yl)-3-methyl-7-oxo-3,7-dihydro-2H-[1,4]oxazino[2,3,4-Ij]quinoline-6-Carboxylic acid (**6e**)*

Yield: 16%; mp 314–315°C; IR (KBr) cm^−1^: 1623 (C = O). ^1^H-NMR (DMSO-*d*_6_): 1.40–1.58 (m, 3H, CH_3_), 2.45-78 (m, 4H, piperazine), 2.85–3.00 (m, 4H, piperazine), 3.51 (s, 2H, CH_2_), 4.35 (d, 1H, CH_2_, *J* = 10.4 Hz), 4.57 (d, 1H, CH_2_, *J* = 10.4 Hz), 4.58–4.49 (m, 1H, CH-Me), 7.20–7.40 (m, 1H, thiophene), 7.56 (d, 1H, H_8_, *J* = 11.6 Hz), 7.66–7.76 (m, 1H, thiophene), 7.82–7.90 (m, 1H, thiophene), 8.96 (s, 1H, H_5_), 11.95 (s, 1H, NOH), 15.20 (s, 1H, COOH).

#### *9-fluoro-10-(4-(2-(methoxyimino)-2-(thiophen-2-yl)ethyl)piperazin-1-yl)-3-methyl-7-oxo-3,7-dihydro-2H-[1,4]oxazino[2,3,4-Ij]quinoline-6-Carboxylic acid (**6f**)*

Yield: 19%; mp 224–225°C; IR (KBr) cm^−1^: 1719 (C = O). ^1^H-NMR (DMSO-*d*_6_): 1.43 (d, 3H, CH_3_, *J* = 4.3 Hz), 2.53–2.63 (m, 4H, piperazine), 3.23–3.31(m, 4H, piperazine), 3.50 and 3.66 (two s, 2H, CH_2_, *E*- and *Z*-isomers), 3.89 (s, 3H, OCH_3_), 4.36 (d, 1H, CH_2_, *J* = 8.8 Hz), 4.57 (d, 1H, CH_2_, *J* = 8.8 Hz), 4.90–4.94 (m, 1H, CH-Me), 7.08–7.23 (m, 1H, thiophene), 7.54 and 7.76 (two d, 1H, thiophene, *E*- and *Z*-isomers, *J* = 4.2 Hz), 7.79 and 7.83 (two d, 1H, thiophene, *E*- and *Z*-isomers, *J* = 4.2 Hz), 7.91 (d, 1H, H_8_, *J* = 10.0 Hz), 8.96 (s, 1H, H_5_), 15.00 (s, 1H, COOH).

#### *10-(4-(2-(benzyloxyimino)-2-(thiophen-2-yl)ethyl)piperazin-1-yl)-9-fluoro-3-methyl-7-oxo-3,7-dihydro-2H-[1,4]oxazino[2,3,4-Ij]quinoline-6-Carboxylic acid (**6g**)*

Yield: 17%; mp 157–158°C; IR (KBr) cm^−1^: 1720 (C = O). ^1^H-NMR (DMSO-*d*_6_): 1.44(d, 3H, CH_3_, *J* = 7.5 Hz), 2.53–2.61 (m, 4H, piperazine), 3.23–3.38 (m, 4H, piperazine), 3.50 and 3.69 (two s, 2H, CH_2_, *E*- and *Z*-isomers), 4.65–4.80 (m, 1H, CH_2_), 4.90–4.97 (m, 1H, CH-Me), 5.17 and 5.28 (two s, 2H, OCH_2_, *E*- and *Z*-isomers), 7.06–7.22(m, 1H, thiophene), 7.30–7.42 (m, 5H, phenyl), 7.52 and 7.59 (d, 1H, thiophene, *J* = 4.4 Hz, *Z*-isomer), 7.77 and 7.89 (two d, 1H, thiophene, *E*- and *Z*-isomers, *J* = 4.4 Hz), 7.94 (d, 1H, H_8_, *J* = 10.0 Hz), 8.96 (s, 1H, H_5_), 15.01 (s, 1H, COOH).

#### *9-fluoro-10-(4-(2-(hydroxyimino)-2-(thiophen-3-yl) ethyl)piperazin-1-yl)-3-methyl-7-oxo-3,7-dihydro-2H-[1,4]oxazino[2,3,4-ij]Quinoline-6-carboxylic Acid (**6h**)*

Yield: 22%; mp 354–356°C; IR (KBr) cm^−1^: 1631, 1719 (C = O). ^1^H-NMR (DMSO-*d*_6_): 1.40–1.48 (m, 3H, CH_3_), 2.53–2.60 (m, 4H, piperazine), 3.37–3.43 (m, 4H, piperazine), 4.31–4.38 (m, 1H, CH_2_), 4.52–4.60 (m, 1H, CH_2_), 4.84–4.92 (m, 1H, CH-Me), 7.50–7.60 (m, 2H, thiophene), 7.72 (d, 1H, thiophene, *J* = 4.5 Hz), 8.38–8.43 (m, 1H, H_8_), 8.94 (s, 1H, H_5_), 11.37 (s, 1H, NOH), 15.19 (s, 1H, COOH).

#### *9-fluoro-10-(4-(2-(methoxyimino)-2-(thiophen-3-yl)ethyl)piperazin-1-yl)-3-methyl-7-oxo-3,7-dihydro-2H-[1,4]oxazino[2,3,4-Ij]quinoline-6-Carboxylic acid (**6i**)*

Yield: 18%; mp 156–157°C; IR (KBr) cm^−1^: 1630, 1721 (C = O). ^1^H-NMR (DMSO-*d*_6_): 1.42 (d, 3H, CH_3_, *J* = 4.8 Hz), 2.51–2.65 (m, 4H, piperazine), 3.24–3.32 (m, 4H, piperazine), 3.52 (s, 2H, CH_2_), 3.91 (s, 3H, OCH_3_), 4.37 (d, 1H, CH_2_, *J* = 8.7 Hz), 4.59 (d, 1H, CH_2_, *J* = 8.7 Hz), 4.91–4.95 (m, 1H, CH-Me), 7.07–7.13 (m, 1H, thiophene), 7.54 (d, 1H, thiophene, *J* = 4.0 Hz), 7.85 (d, 1H, thiophene, *J* = 4.0 Hz), 7.90 (d, 1H, H_8_, *J* = 10.3 Hz), 8.98 (s, 1H, H_5_), 15.20 (s, 1H, COOH).

#### *10-(4-(2-(benzyloxyimino)-2-(thiophen-3-yl) ethyl) piperazin-1-yl)-9-fluoro-3-methyl-7-oxo-3, 7-dihydro-2H-[1,4]oxazino[2,3,4-ij]quinoline-6-carboxylic acid (**6j**)*

Yield: 17%; mp 162–163°C; IR (KBr) cm^−1^: 1621, 1716 (C = O). ^1^H-NMR (DMSO-*d*_6_): 1.43 (d, 3H, CH_3_, *J* = 6.8 Hz), 2.40–2.67 (m, 4H, piperazine), 3.17–3.44 (m, 4H, piperazine), 3.54 and 3.65 (two s, 2H, CH_2_, *E*- and *Z*-isomers), 4.54–4.58 (m, 1H, CH_2_), 4.90–4.93 (m, 1H, CH-Me), 5.14 and 5.28 (two s, 2H, OCH_2_, *E*- and *Z*-isomers), 7.06–7.20 (m, 1H, thiophene), 7.39 (d, 1H, thiophene, *J* = 4.0 Hz, *Z*-isomer), 7.44 (d, 1H, thiophene, *J* = 4.0 Hz, *Z*-isomer), 7.48–7.68 (m, 5H, phenyl), 7.68 (d, 1H, thiophene, *J* = 4.0 Hz, *E*-isomer), 8.31 (d, 1H, thiophene, *J* = 4.0 Hz, *Z-*isomer), 8.38 (d, 1H, thiophene, *J* = 4.4 Hz, *E*-isomer), 8.01 (s, 1H, H_8_), 8.96 (s, 1H, H_5_), 15.01 (s, 1H, COOH).

### Antimicrobial activity

Compounds **6a–j** were screened for their antibacterial activity against Gram-positive (*Staphylococcus aureus* ATCC 6538, *Staphylococcus epidermidis* ATCC 12228, *Bacillus subtilis* ATCC 6633) and Gram-negative (*E. coli* ATCC 8739, *Pseudomonas aeruginosa* ATCC 9027, *Klebsiella pneumoniae* ATCC 10031) bacteria by the conventional agar dilution method [18]. Two-fold serial dilutions of the compounds and reference drugs (levofloxacin and *N*-desmethyl levofloxacin) were prepared in Mueller-Hinton agar. Drugs (10.0 mg) were dissolved in DMSO (1 mL) and the solution was diluted with water (9 mL). Further progressive double dilution with melted Mueller-Hinton agar was performed to obtain the required concentrations of 100, 50, 25, 12.5, 6.25, 3.12, 1.56, 0.78, 0.39, 0.19, 0.09, 0.04, 0.02 and 0.01 μg/mL. Petri-dishes were inoculated with 1–5 × 10^4^ colony-forming units (cfu) and incubated at 37°C for 18 h. The minimum inhibitory concentration (MIC) was defined as the lowest concentration of the test compound, which resulted in no visible growth on the plate. To insure that the solvent had no effect on bacterial growth, a control test was performed with test medium supplemented with DMSO at the same dilutions as used in the experiment.

## Results and discussion

As summarized in Figure 2, the *N*-substituted analogs of levofloxacin **6a–j **were prepared by nucleophilic reaction of *N*-desmethyl levofloxacin **11** with thienylethyl bromide derivatives **8** or **9** employing reaction sequences previously described by us for the preparation of other *N*-substituted piperazinyl quinolones. Thus, the oxime derivatives **9** were synthesized by the reaction of ketone **8** with hydroxylamine hydrochloride or *O*-methylhydroxylamine hydrochloride or *O*-benzylhydroxylamine hydrochloride [12,14]. On the other hand, the piperazinyl quinolone (**11**, *N*-desmethyl levofloxacin) was prepared according to the known method [17], by the reaction of piperazine with 9,10-difluoro-2,3-dihydro-3-methyl-7-oxo-7H-pyrido[1,2,3-de][1,4]benzoxazine-6-carboxylic acid **10**. Reaction of *N*-desmethyl levofloxacin **11** with thienyl ethyl bromide derivatives **8** or **9** in DMF, in the presence of NaHCO_3_ at room temperature afforded corresponding ketones **6a,b** and oxime derivatives **6c–j**, respectively.

 The minimum inhibitory concentrations (MICs, μg/mL) obtained for compounds **6a–j** are presented in Table 1. In general, Table 1 reveals that higher susceptibilities were observed with Gram-positive and poorer susceptibilities with Gram-negative bacteria. The MIC values of ketones **6a,b** and oximes **6c–j** against Gram-positive strains indicate that most compounds possessed a comparable or better activity (MIC = 0.04–3.12 μg/mL) with respect to *N*-desmethyl levofloxacin **11** and levofloxacin **4** (MIC = 0.25–4 μg/mL). As is evident from the data, oximes **6e**, **6h** and **6i** followed by ketone **6a** are superior in inhibiting the growth of Gram-positives (MIC = 0.04–0.19 μg/mL), and their activities were found to be 5–25 times better than *N*-desmethyl levofloxacin **11** and equal or better than levofloxacin **4**. In general, most compounds showed weak to good activity (MIC = 1.56–100 μg/mL) against Gram-negative bacteria. Compound **6b** was the most potent against *E. coli*, *Pseudomonas aeruginosa* and *Klebsiella pneumonia* (MIC = 1.56–6.25 μg/mL). Comparison between MIC values of 2-thienyl drivatives **6e–g** and 3-thienyl compounds **6h–j** revealed that the corresponding regioisomers have a same antibacterial activity and this modification cannot improve the activity. Also, etherification of oximes by methyl or benzyl groups dramatically diminished the activity against Gram-negatives. However, by *O*-methylation, the activity against Gram-positive bacteria was maintained. In addition, by comparison of 5-chlorothiophene derivatives **6c,d** and corresponding unsubstituted analogs **6f,g** it could be concluded that halogen substituent is not important for activity.

**Table 1:** In vitro antibacterial activities (MICs in μg/mL) of compounds 6a–j


**Compound**	**R**	**Y**	***S. aureus***	***S. epidermidis***	***B. subtilis***	***E. coli***	***P. aeruginosa***	***K. pneumoniae***
**6a**		O	0.19	0.19	0.19	12.5	100	100
**6b**		O	0.39	0.19	0.09	1.56	6.25	1.56
**6c**		NOCH_3_	0.78	0.39	0.19	50	50	12.5
**6d**		NOBn	3.125	1.56	0.78	50	100	100
**6e**		NOH	0.19	0.19	0.19	3.125	25	3.125
**6f**		NOCH_3_	0.39	0.19	0.09	50	100	25
**6g**		NOBn	6.25	3.125	0.78	100	100	50
**6h**		NOH	0.19	0.09	0.04	3.125	12.5	3.125
**6i**		NOCH_3_	0.19	0.19	0.09	100	100	50
**6j**		NOBn	6.25	3.125	0.39	50	100	25
**Levofloxacin**			0.25	0.25	0.5	0.03	4	0.25
***N*-Desmethyl levofloxacin**			4	1	1	0.012	>4	0.25

## Competing interests

The author(s) declare that they have no competing interests.

## Authors' contribution

NM: Synthesis of some target compounds (15%). ZA: Synthesis of the intermediates and some target compounds (10%). EA: Collaboration in identifying of the structures of target compounds (10%). SE: Collaboration in design and identifying of the structures of target compounds, manuscript preparation (10%). MAF: Evaluation of the antibacterial activities (10%). NS: Evaluation of the antibacterial activities (10%). NK: Evaluation of the antibacterial activities (5%). AS: Collaboration in identifying of the structures of target compounds (10%). AF: Design of target compounds and management of the synthetic and pharmacological parts (20%). All authors read and approved the final manuscript.
